# Molecular Characterization of Carbapenem and Colistin Resistance in Isolates Causing Urinary Tract Infections at an Outpatient Setting of a Tertiary Care Hospital

**DOI:** 10.7759/cureus.98941

**Published:** 2025-12-10

**Authors:** Chandan Mishra, Suneeta Meena, Purva Mathur

**Affiliations:** 1 Laboratory Medicine, All India Institute of Medical Sciences, New Delhi, New Delhi, IND; 2 Microbiology, All India Institute of Medical Sciences, New Delhi, New Delhi, IND

**Keywords:** carbapenemase genes, carbapenem resistant gram negative infection, ca-uti, colistin resistance, community acquired uti

## Abstract

Background

Increasing resistance to higher-end antibiotics like carbapenems and colistin is a great cause of concern. There is a paucity of data on the detection of carbapenem and colistin in outpatient settings. This study was to determine the prevalence of carbapenem-resistant Enterobacterales (CRE) and colistin resistance in CRE at the outpatient setting of a tertiary care institute of North India.

Methods

A prospective study was conducted from January to December 2020. A total of 6,829 non-repetitive urine samples were cultured on cysteine lactose electrolyte-deficient (CLED) agar. Identification was done by matrix-assisted laser desorption ionization-time of flight mass spectrometry (MALDI-TOF MS), and antimicrobial susceptibility testing followed Clinical and Laboratory Standards Institute (CLSI) 2020, using Kirby-Bauer and Vitek 2 (BioMérieux, Marcy-l'Étoile, France). Carbapenem-resistant isolates were screened for carbapenemase genes (*bla_NDM_*, *bla_KPC_*, *bla_OXA_*, *bla_OXA-48_*). Colistin resistance was confirmed by broth microdilution.

Results

A total of 669/6829 (9.79%) urine specimens yielded significant growth, mostly in females (54.1%), less than in other studies probably due to fewer samples received because of the COVID-19 pandemic. The majority (50.82%) of patients belonged to the early adult group (15-45 years). Most organisms were *Enterobacteriaceae* (509/669, 76.08%). The most common organism isolated was *E. coli* (56.5%) followed by *K. pneumoniae *(18.2%). 5.97% (40/669) isolates were carbapenem-resistant. 6.87% (35/509) of *Enterobacteriaceae* were carbapenem-resistant, which is less than in other studies possibly due to samples from outpatient settings. The following carbapenemase genes were detected: *bla_NDM_* (18/35),* bla_KPC_* (2/35), *bla_OXA _*(11/35) and *bla_OXA-48_* (5/35). Some isolates had co-existence of more than two carbapenemase genes. Colistin resistance in CRE was 11% which is like other studies conducted in the same tertiary centre. Comparison of resistance patterns of other antibiotics in CRE and carbapenem-sensitive Enterobacterales (CSE) group shows that fosfomycin, ticarcillin-clavulanate and tetracycline had higher sensitivity in the CRE group.

Conclusion

The presence of these genes in outpatient settings is worrisome. Both *bla*_*NDM* _and *bla_OXA-48_* resulted in higher minimum inhibitory concentrations (MICs) against carbapenems. Co-presence of *NDM* with *OXA-48*-producing *E. coli *in urine culture. Colistin resistance in an outpatient setting is alarming. Early detection of these resistance-determinant genes by molecular methods is essential in limiting the spread of infection due to these organisms.

## Introduction

Antimicrobial resistance has challenged the treatment of common infections and is a threat to public health systems [1]. Carbapenems and colistin are the “last resort” antibiotics for treating infections caused by extended-spectrum β-lactamase (ESBL)-producing Enterobacteriaceae [2].

Carbapenems, a beta-lactam group of antibiotics, kill bacteria by binding to the penicillin-binding proteins, inhibiting bacterial wall synthesis. Carbapenems are broad-spectrum antibiotics against gram-negative organisms and have a somewhat narrower spectrum against gram-positive organisms. Carbapenems are critically important drugs, so resistance to these is a serious healthcare concern [3].

Carbapenem-resistant Enterobacteriaceae (CRE) are defined by resistance to at least one carbapenem or the presence of a carbapenemase. CRE can be carbapenemase-producing (CP-CRE) or non-carbapenemase-producing CRE (non-CP-CRE) strains. CP-CRE produce carbapenemases enzymes to hydrolyse carbapenem, whereas non-CP-CRE have *β*-lactamase (ESBLs and AmpC enzymes) activity combined with structural mutations of membrane protein and efflux pumps [4].

After development of CRE there are few options left for treatment of multidrug-resistant (MDR) as well as extensively drug-resistant (XDR) gram-negative bacteria. Knowing risk factors associated with CRE acquisition in patients is important, as it has implications for empirical antibiotic therapy and also benefits in infection control. The emergence of CRE among populations is a serious threat to proper antibiotic therapy and eradication of infection. It requires the implementation of multilayered strategies and meticulous surveillance in healthcare and community settings. Carbapenems need to be prescribed and used cautiously [5].

Antimicrobial resistance to carbapenems that has become prevalent in inpatient settings is creeping into the community. In our laboratory we receive urine samples from outpatients for antimicrobial susceptibility. This study setting contains a mix of both inpatients who are followed up and patients from the community. Hence, this study reports the status of antimicrobial resistance in the outpatient setting in our hospital.

This article was previously presented as a meeting abstract at the 2025 ADLM Annual Scientific Meeting on July 29, 2025 [6].

## Materials and methods

This is a prospective observational cohort study conducted in the Microbiology section of the Department of Laboratory Medicine, All India Institute of Medical Sciences (AIIMS), New Delhi. Ethical approval was obtained from the Institutional Ethics Committee prior to the start of the study (Ref. No.: IECPG-761/30,01.2020). During the period of study, freshly voided midstream urine samples (10-15 ml) collected in a sterile container with screw cap tops were accepted from different OPDs and processed in the lab within one to two hours for analysis. Urine samples were examined chemically and microscopically. Out of 567 gram-negative bacterial isolates, 43 that were resistant to carbapenem i.e., ertapenem, imipenem or meropenem were selected.

Organisms were identified using matrix-assisted laser desorption ionization-time of flight mass spectrometry (MALDI-TOF MS). A mass spectrum of each organism was generated and automatically compared against a database of mass spectra by MYLA® software, resulting in the identification of the organism. Quality control strain Escherichia coli ATCC-8739 was used to calibrate the instrument for each run.

Antimicrobial susceptibility testing used Vitek 2 (BioMérieux, Marcy-l'Étoile, France) and reported as per Clinical and Laboratory Standards Institute (CLSI) guidelines 2020. Antimicrobial susceptibility results were expressed in terms of measure of accuracy. Results of susceptibility tests were categorized as susceptible (S), intermediate (I), or resistant (R) according to criteria recommended by CLSI guidelines.

The bacterial isolates from the urine samples were subjected to DNA extraction by using HiPurA Genomic DNA Purification Kit (HiMedia Laboratories Pvt., Thane, India). The standard method as given in the kit insert was used for the bacterial DNA extraction. The extracted bacterial DNA was subjected to amplification of the specific region of different genes (NDM, KPC, OXA-1, OXA-48) using primers from Integrated DNA Technologies (Coralville, IA, USA) (Appendix).

Statistical analysis

Data was analysed using Stata statistical software, version 15 (StataCorp 2017, College Station, TX, USA), RStudio (Posit PBC, Boston, MA, USA) software version 2025.05.0+496 and Microsoft Excel 2019 (Redmond, WA, USA) were used for data analysis and graphical representation. Wilcoxon rank sum test, Pearson’s Chi-squared test, and Fisher’s exact test were used for calculation of statistical significance.

**Figure 1:**
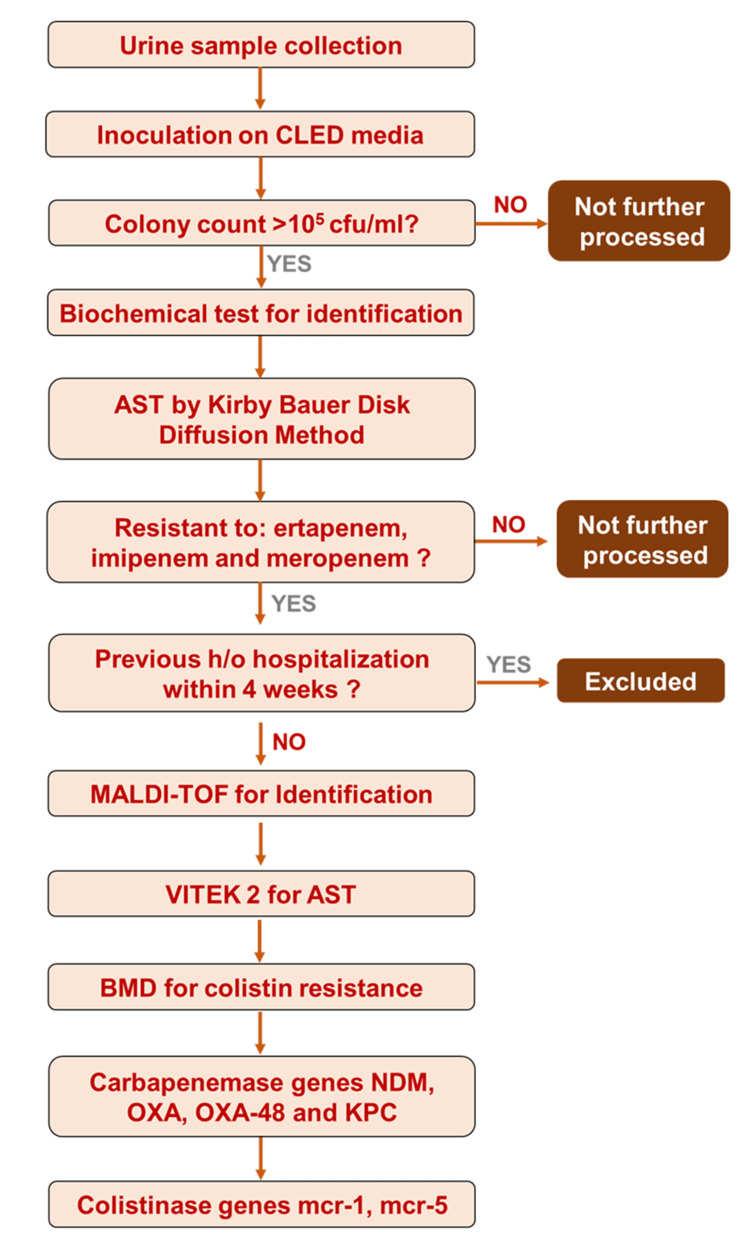
Flowchart of Methodology CLED - cysteine lactose electrolyte deficient medium cfu - colony forming unit AST - Antibiotic Sensitivity Testing h/o - history of MALDI TOF - Matrix-Assisted Laser Desorption Ionization–Time of Flight BMD - Broth Micro Dilution mcr-1 - mobilized colistin resistance -1 mcr-5 - mobilized colistin resistance -5

The flow of the study is given in Figure 1.

## Results

**Figure 2:**
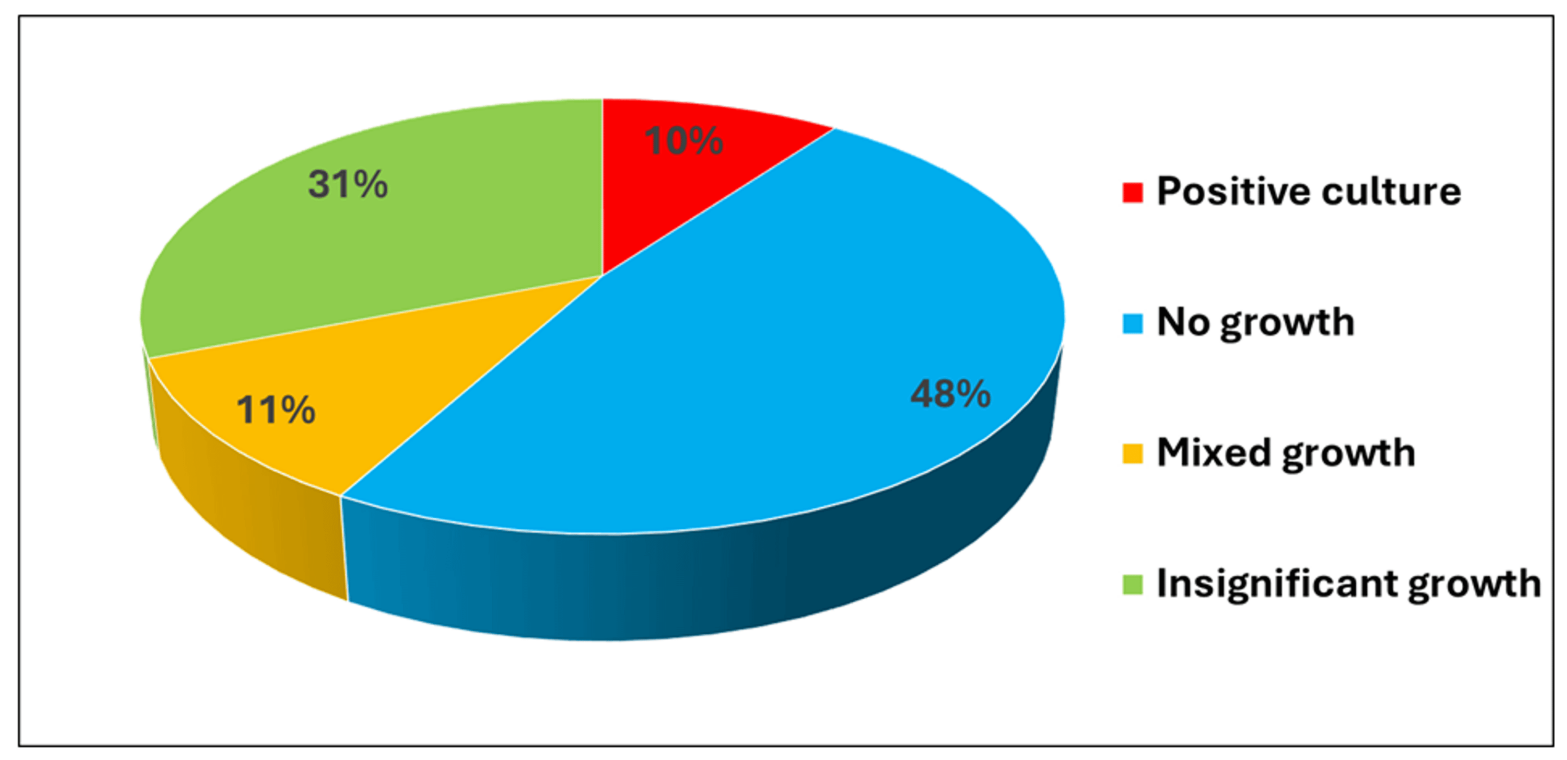
Urine specimen yields for culture growths.

**Figure 3:**
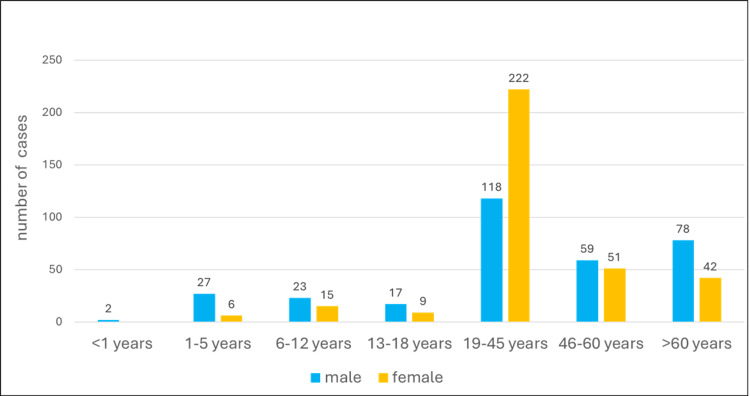
Age group-wise population distribution.

**Figure 4:**
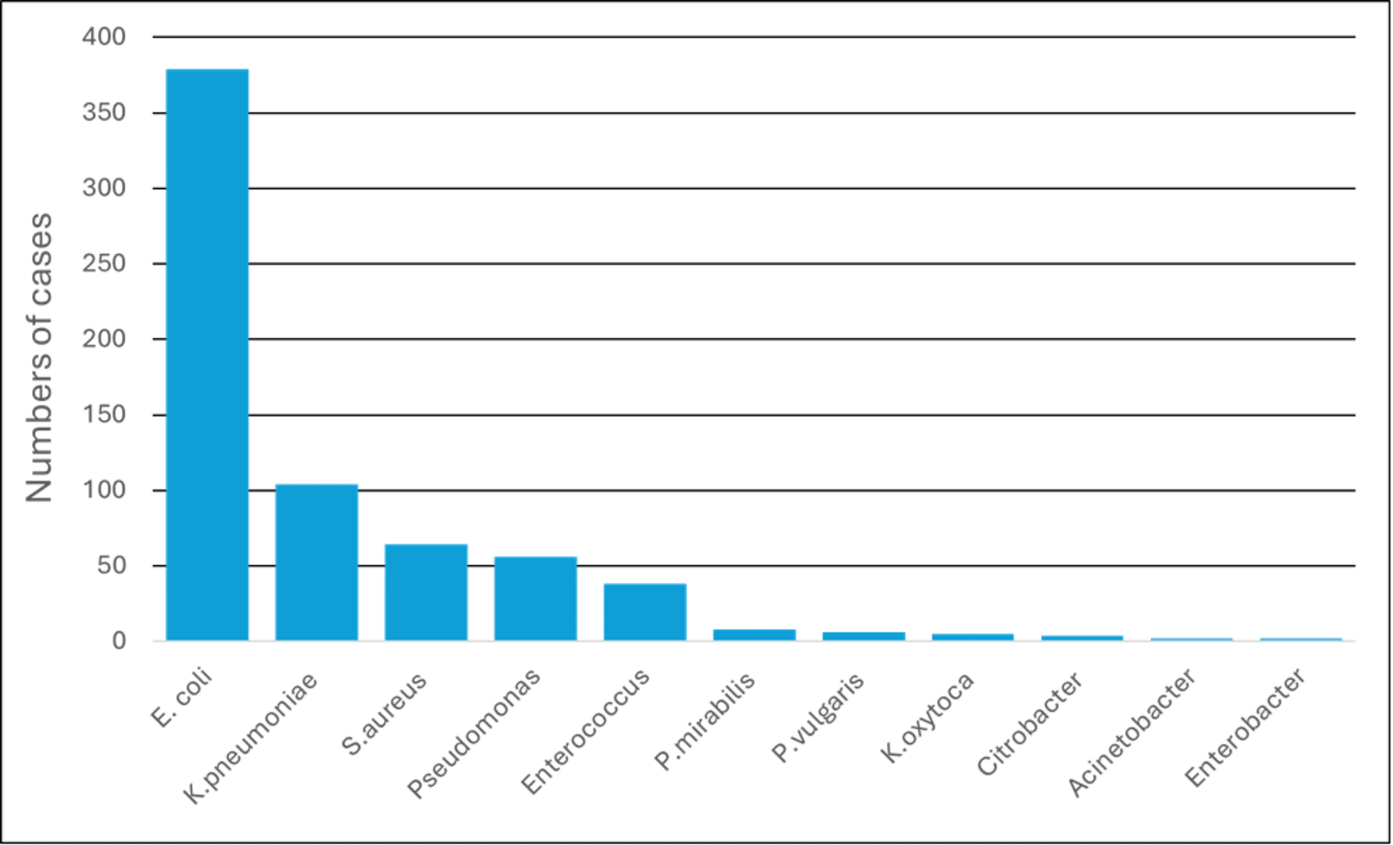
Urinary isolates-wise distribution.

In this study a total of 6829 urine samples were collected between 1st January 2020 to 31st December 2020. Of 6829 samples, 669 were positive for the growth of pathogenic strains after removing duplicates (Figure 2). The majority (50.82%) of patients belonged to the early adult group (15-45 years) (Figure 3). Among the total pathogenic isolates, the Gram-negative isolates were most representative with 567 and were included in the study. Gram-negative isolates comprised 379 *Escherichia coli* (*E. coli*), 104 *Klebsiella pneumoniae* (*K. pneumoniae*), 56 *Pseudomonas aeruginosa* (*P. aeruginosa*), eight *Proteus mirabilis* (*P. mirabilis*), six *Proteus vulgaris*, five *Klebsiella oxytoca* (*K. oxytoca*), four *Citrobacter *spp., two *Acinetobacter *spp., two *Enterobacter *spp., and one *Morganella *sp. (Figure 4). Out of 567 Gram-negative bacterial isolates, 509 were enterobacterial, of which 35 were identified as CRE. The characteristics of these CRE and CSE are given in Table 1.

**Table 1:** Characteristics of CRE and CSE CRE - Carbapenem Resistant Enterobacteriaceae CSE - Carbapenem Sensitive Enterobacteriaceae ^1^ Median (Q1, Q3); n (%) *^2^* Wilcoxon rank sum test *^3^* Pearson’s Chi-squared test *^4^* Fisher’s exact test

Characteristics	Overall (n=509^1^)	CRE (n=35^1^)	CSE (n=474^1^)	p-value
Age	37 (25, 54)	32 (14, 46)	38 (25, 55)	0.2^2^
Gender				
F	263 (52%)	12 (34%)	251 (53%)	0.009^3^
M	246 (48%)	23 (66%)	223 (47%)	
Organism				
E.coli	379 (74%)	23 (65%)	356 (75%)	0.6^4^
K.pneumoniae	104 (20%)	10 (29%)	93 (20%)	
P.mirabilis	8 (1.6%)	-	7 (1.5%)	
P. vulgaris	6 (1.2%)	-	6 (1.3%)	
K. oxytoca	5 (1.0%)	-	5 (1.1%)	
Citrobacter spp.	4 (0.8%)	-	4 (0.8%)	
Enterobacter spp.	2 (0.4%)	1 (3%)	1(0.2%)	
Morgenella spp.	1 (0.2%)	1 (3%)	-	

Carbapenemase in carbapenem-resistant Enterobacteriaceae

**Figure 5:**
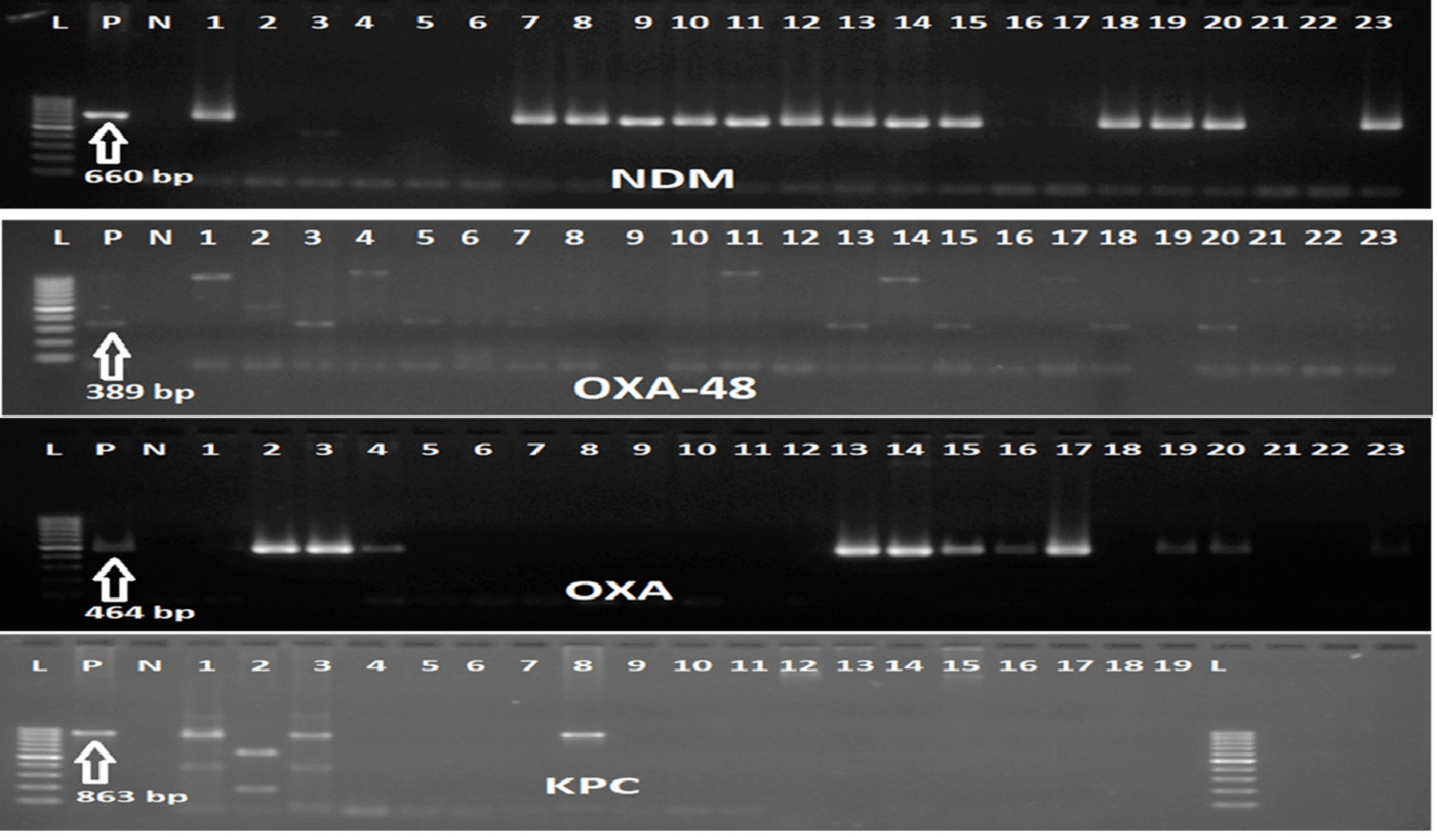
Gel electrophoresis for desired gene amplicon. The arrow indicates positive (P) control for the concerned gene. (L) is ladder. (N) is negative control. *KPC* - Klebsiella pneumoniae carbapenemase *NDM* - New Delhi Metallo-β-lactamase *OXA-48* - Oxacillinase-48 *OXA* - Oxacillinase

**Figure 6:**
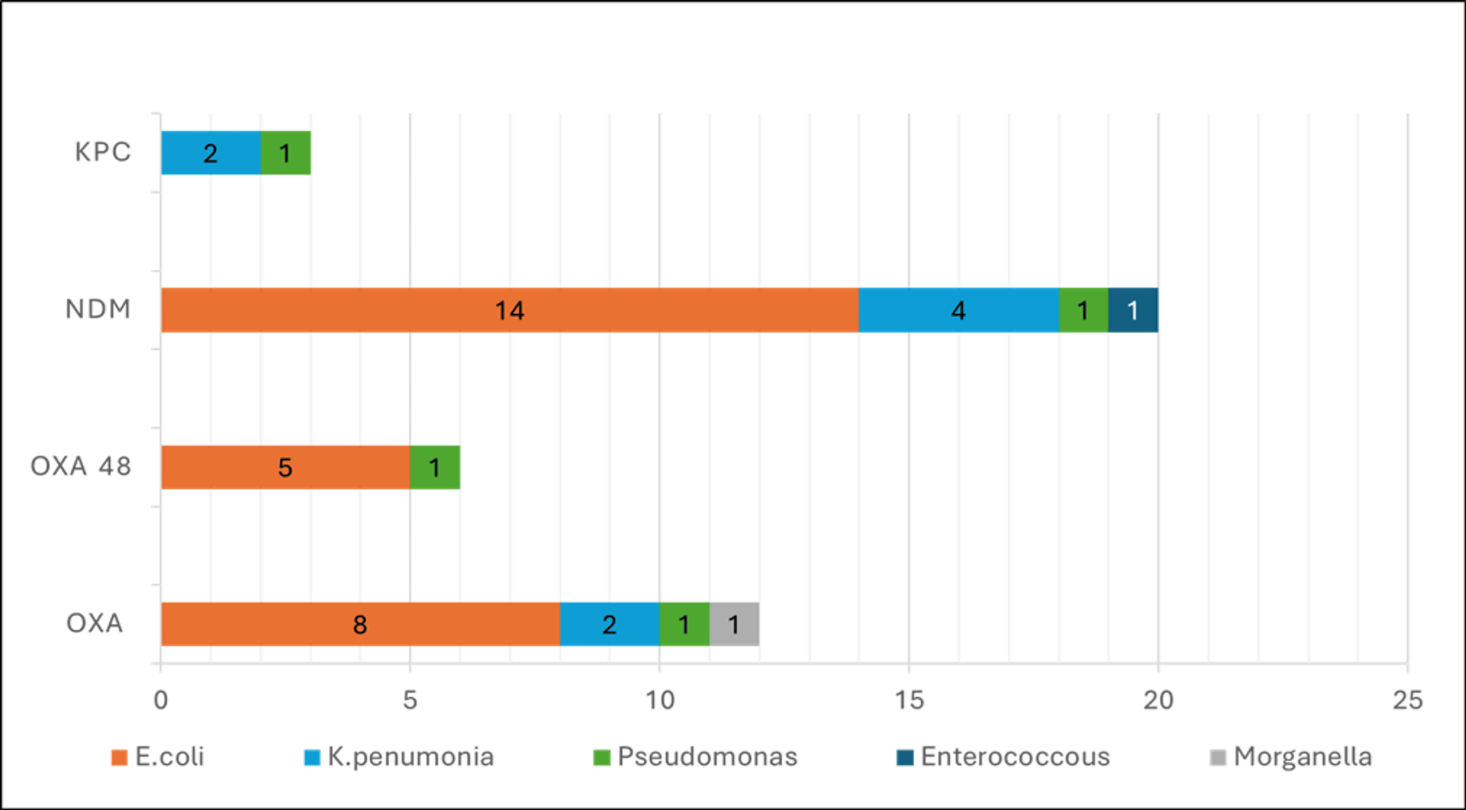
Gene representation of carbapenamase. *KPC* - Klebsiella pneumoniae carbapenemase *NDM* - New Delhi Metallo-β-lactamase *OXA-48* - Oxacillinase-48 *OXA* - Oxacillinase

**Figure 7:**
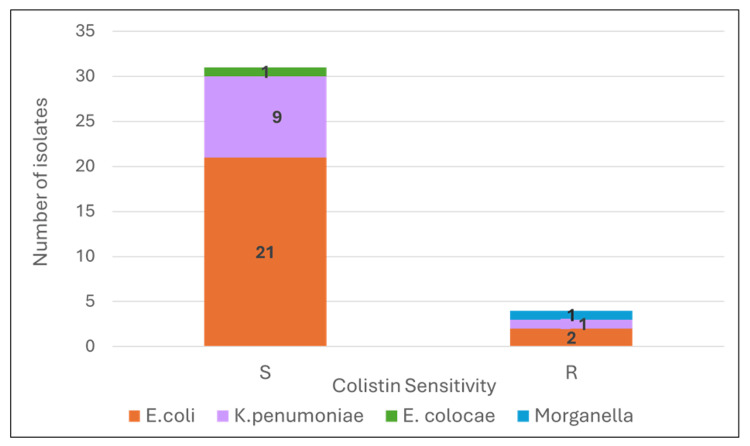
BMD for colistin resistance in CRE. BMD - Broth Micro Dilution E. coli - Escheriachae coli K. pneumoniae - Klebsiella pneumoniae E. cloacae - Enterobacter cloacae S - Sensitive for colistin R - Resistant for colistin

Polymerase chain reaction (PCR) was performed for detection of carbapenemase and colistin resistance genes. The presence of carbapenemase was determined by the identification of four genes: *bla_NDM_*, *bla_KPC_*, *bla_OXA_*, *and bla_OXA-48_* (Figure 5). A total of 35 carbapenem-resistant enterobacterials were identified by disk diffusion method and verified by Vitek 2. The organisms identified were *E. coli* (n=21), *Klebsiella pneumoniae* (n=10), *Pseudomonas aeruginoas* (n=6), *Acinetobacter baumanii* (n=1), *Morganella morganii* (n=1) and *Enterobacter cloacae* (n=1). The most prevalent carbapenemase gene was *NDM* (18/35), followed *by OXA* (11/35), *OXA-48* (5/35) and *KPC* (2/40). Some isolates had co-existence of two or more carbapenemase genes; four *E. coli*, one *K. pneumoniae*, and one *Pseudomonas* presented with *OXA* and *OXA-48* genes, one *E. coli* with *OXA* and *NDM* genes, one *K. pneumoniae* with *NDM* and *KPC* genes, and two *E. coli* with *OXA*, *OXA-48*, and *NDM* genes. The* NDM* gene was present in 14 *E. coli*, four *K. pneumoniae* and one *Enterobacter*. The* OXA* gene was present in eight *E. coli*, two *K. pneumoniae*, and one *Morganella morganii*. *OXA-48* was seen in five *E. coli*. *KPC* was in two *K. pneumoniae* (Figure 6). Those resistant to carbapenem were tested for colistin resistance by broth microdilution (BMD). Colistin resistance among CRE was 11% (Figure 7).

We also compared the antibody resistance pattern in the CRE and CSE. This comparison revealed that all antibiotics showed significant results except fosfomycin, ticarcillin/clavulanate and tetracycline (Table 2).

**Table 2:** Comparison of other antibiotics between CRE and CSE groups. *^1^* Fisher’s exact test *^2^* Pearson’s Chi-squared test * significant ** very significant CRE - Carbapenem Resistant Enterobacteriaceae CSE - Carbapenem Sensitive Enterobacteriaceae AK=amikacin, AMC=amoxicillin-clavulanic acid, AMP=ampicillin, AS=ampicillin-sulbactam, AT=aztreonam, CAZ=ceftazidime; CIP=ciprofloxacin, COT=cotrimoxazole, CPM=cefepime, CX=cefoxitin, CZ=cefazolin, FO=fosfomycin, GEN=gentamycin, LE=levofloxacin, NIT=nitrofurantoin, NX=norfloxacin, PIT=piperacillin-tazobactam; TCC=ticarcillin-clavulanic acid; TE=tetracycline, TOB=tobramycin

Antibiotic	CRE n,(%)	CSE n,(%)	p-value
AK	14 (78%)	3 (38%)	0.078*^1^*
AMC	35(100%)	395 (87%)	0.015^1^*
AMP	35 (100%)	378 (83%)	0.007^2^*
AS	31 (89%)	199 (45%)	<0.001^2^**
AT	15 (100%)	1 (25%)	0.004^1^*
CAZ	21 (100%)	51 (59%)	<0.001^2^**
CIP	35 (100%)	342 (75%)	<0.001^2^**
COT	31 (89%)	249 (57%)	<0.001^1^**
CPM	17 (94%)	11 (69%)	0.078^1^
CX	30 (88%)	180 (41%)	<0.001^1^**
CZ	28 (97%)	153 (69%)	0.002^2^*
FO	11 (31%)	47 (10%)	0.001^1^*
GEN	17 (94%)	27 (26%)	<0.001^2^**
LE	33 (94%)	331 (74%)	0.016^1^*
NIT	20 (59%)	97 (21%)	<0.001^1^**
NX	6 (100%)	27 (56%)	0.071^1^
PIT	15 (88%)	4 (44%)	0.028^1^*
TCC	18 (100%)	4 (67%)	0.054^1^
TE	28 (80%)	208 (47%)	<0.001^2^**
TOB	16 (100%)	2 (40%)	0.008^1^*

## Discussion

Antimicrobial resistance (AMR) is a worrisome epidemic that is slowly engulfing the whole world. Simple cases of uncomplicated UTI are also becoming difficult to manage due to AMR [7-9]. However, the burden of UTI and AMR may vary in different settings. UTI being the most common infection in the outpatients [10], and the second most common infection for an antimicrobial prescription (after respiratory infection) [11], this study was conducted to find the burden of carbapenem resistance in these patients.

Our study highlighted the prevalence of UTI and AMR in an outpatient setting, which has not been addressed in many other studies before. It was a prospective observational study, conducted over 12 months. A total of 6829 urine samples had 9.79% (669) pathogenic isolates. Sample positivity was also lower compared with the studies from North India [12]. The study was conducted during the COVID-19 pandemic when there was an upsurge of telemedicine (a remote provision of clinical care), where common ailments were treated by empirical therapy and also the outpatient service were running in limited capacity, which might also have affected the outcome of the study.

Microbial profile of uropathogens

In our study, the gram-negative organisms were 84.75%, gram-positive 15.25%. *Escherichia coli* (56.65%) was most prevalent among gram-negative organisms. This result was consistent with other studies from India [9,13,14]. The result was also consistent with studies from Africa [15], Europe [16], the Middle East [17], and Caribbean countries [18].

Other organisms included were* K. pneumoniae* (15.54%), *S. aureus* (9.56%), *P. aeruginosa* (8.37%), *Proteus *spp*.* (1.19%), and *Enterobacter *spp*.* (0.29%). Similar to our study other studies also showed *Klebsiella *spp. as the second most frequently isolated organism in UTI [19-23].

The studies on UTI done in other parts of the world also showed that *E. coli* and *Klebsiella *spp. are the commonest uropathogens in UTI [13,24-26]. *Enterobacteriaceae* have several virulent factors that enable them to cause UTI. They can easily attach to the uroepithelium of urogenital mucosa by adhesins, pili, fimbriae, and P-1 blood group phenotype receptors [27].

Carbapenemase in carbapenem-resistant Enterobacteriaceae

PCR was done for the detection of carbapenemase-producing genes such as *bla*_KPC_, *bla*_oxa_, *bla*_NDM_, and *bla*_OXA-48_. The most prevalent carbapenemase gene was *NDM* (20/40), followed by *OXA* (12/40), *OXA-48* (6/40), and *KPC* (3/40). The* NDM* gene was present in 14 *E. coli* isolates, four *K. pneumoniae* isolates, one *Pseudomonas* and one *Enterobacter*. The *OXA* gene was present in eight *E. coli* isolates, two *K. pneumoniae*, one *Morganella morganii*, and one *Pseudomonas aeruginosa*. *OXA-48* was seen in five *E. coli* and one *Pseudomonas*. *KPC* was found in two *K. pneumoniae* and one *Pseudomonas* [28].

Similar findings were seen in other studies from India, where the *NDM* gene was seen in most of the carbapenem-resistant isolates, followed by *OXA-48* [29,30]. Whereas in some studies *OXA-48* gene was most prevalent followed by *NDM*, wherein the authors have suggested that *OXA-48* is becoming the most prevalent carbapenemase gene in *K. pneumoniae* [27].

In our study some isolates had co-existence of two or more carbapenemase genes. Four *E. coli*, one *K. pneumoniae* and one *Pseudomonas* presented with *OXA* and *OXA-48* genes, one *E. coli* with *OXA* and *NDM* genes, one *K. pneumoniae* with *NDM* and *KPC* genes and two *E. coli* with *OXA*, *OXA-48* and *NDM* genes. This similar co-existence of carbapenamase genes has also been reported in other studies [28-30]. Nine isolates did not show any of the carbapenemase out of four genes that were detected. which can be due to structural cause (porin loss) or hyperproduction of AmpC beta-lactamase or other carbapenemases [4].

Limitations

The sample size was small and the samples collected were during the COVID pandemic. A large-scale study is required in this region. All known causes of carbapenem resistance and colistin resistance were not included; thus, all potential resistance mechanisms were not included due to time and budget constraints. If modern sequencing-based molecular techniques could be used it would facilitate precise detection of all mechanisms of underlying carbapenem and colistin resistance. 

## Conclusions

Against the background of paucity of reports on prevalence of carbapenem and colistin resistance in uropathogens in an outpatient setting, this is the first study to determine the resistance in Enterobacteriaceae for last resort antibiotic i.e., carbapenems by molecular methods in the community. This study also fills the void in determining the epidemiology of uropathogens and antimicrobial resistance by conventional and automated methods in the outpatient setting. Our study showed that ampicillin, amoxicillin/clavulanic acid, ciprofloxacin, levofloxacin, cefoxitin, cefepime and cotrimoxazole had resistance >50%. Carbapenem-resistant gram-negative bacilli were 7.47%, which is a significant level in a community setting. Overall, we observed carbapenem-resistant isolates had carbapenemase-producing genes *NDM*, *KPC*, *OXA* and *OXA-48*. Some *E. coli* had the three carbapenemase genes (*NDM*, *KPC* and *OXA*). 

Thus, we conclude community isolates are also showing multi-drug resistance to antibiotics and even against the last resort antibiotics. Carbapenem resistance that was prevalent in inpatient settings is now creeping into the community. Multiple studies with a larger sample should be conducted for carbapenem and colistin resistance in the region. It is recommended that empirical antimicrobial treatment should be based on the localized epidemiological trend. Our study reports status of antimicrobial resistance in the outpatient setting in our hospital, in order to establish guidelines for the correct use of antimicrobials. Therefore, action is needed from a broad range of stakeholders including clinicians, laboratory physicians and public health officers to limit the spread of antimicrobial resistance.

## Figures and Tables

**Figure 8:**
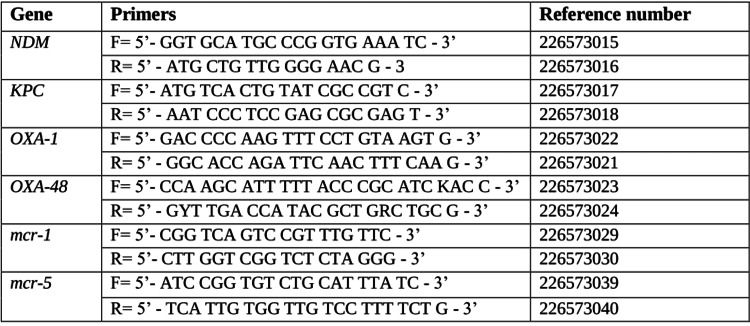
Primers (Integrated DNA Technologies)

## Appendices

Bacterial DNA Extraction

 The bacterial isolates from the urine samples were subjected to DNA extraction by using HiPurA Genomic DNA Purification Kit (HiMedia Laboratories Pvt.). Following steps were involved for the Bacterial DNA extraction.

1. In a 100ml normal saline, taken in a 400ml plastic tube, 2 loops (3 mm diameter) full of bacterial colonies from growth media (kept overnight in an incubator) were added.

2. After adding the tube was vortexed for 15-20 seconds to make a homogenous suspension. In a 2ml aliquot, 200µL suspension was taken.

3. Then 20µL Proteinase K (20mg/ml) enzyme was added to the aliquot and vortexed for 15-20 seconds to ensure thorough mixing.

4. Then 20µL RNAase A solution (20mg/ml) was added to the aliquot to make RNA-free genomic DNA and vortexed for 15-20 seconds.

5. After that 200µL of Lysis solution was added to the suspension, vortexed thoroughly for a few seconds to make a homogenous mixture.

6. Then the aliquot was incubated in a prewarmed water bath at 56°C for 10 minutes.

7. Then 200µL ethanol (96%) was added to the lysate obtained after 10 minutes of incubation. It was thoroughly mixed by gentle pipetting to make a homogenous solution.

8. Then the lysate was loaded in the spin column (capped) placed in a collection tube using a large-bore pipette and centrifuged at 10,000 rpm (≈6,500g) for 1 minute.

9. The flow-through liquid was discarded along with the collection tube and the column was placed in a similar 2.0ml collection tube.

10. 500µL diluted Prewash solution (diluted as indicated in general preparation instructions) was added to the column and again centrifuged at 10,000 rpm (≈6,500g) for 1 minute.

11. The flow-through liquid was discarded along with the collection tube. The column was again placed in a similar collection tube.

12. 500µL of diluted Wash solution (diluted as indicated in general preparation instructions) was added and centrifuged at 13,000 rpm (≈12,000g) for 3 minutes.

13. The flow-through liquid was discarded along with the collection tube.

14. Now the column was placed in a DNA collection tube (capped) and 100µL of the Elution Buffer was added directly into the column without spilling to the sides and incubated for 1 minute at room temperature (15ᵒC - 25ᵒC).

15. Then it was centrifuged at 10,000 rpm (≈6,500g) for 1 minute to elute the DNA. Then it was again repeated with 100µL of Elution Buffer for a high yield of DNA.

16. The column was discarded, and the DNA collection tube eluate that contains pure genomic DNA was labelled and stored at -80°C for further use.

Polymerase Chain Reaction

The extracted Bacterial DNA was subjected to amplify the specific region of different genes (NDM, KPC, OXA-1, OXA-48, mcr-1 and mcr-5) using primers (Integrated DNA Technologies) represented below in the table.

Steps of PCR

1. The PCR was performed by taking 20µL Master mix that contained 14.25 µL nuclease-free water, 2.5µL reaction buffer, 0.5µL of MgCl2, 0.5µL of each forward primer, reverse primer, and dNTPs, along with 0.25µL of Taq polymerase.

2. After the addition of 5µL extracted bacterial DNA, the final volume of the content of the PCR tube was 25µL.

3. PCR conditions were standardized as follows:

**Table 3:** PCR conditions

Gene	Denaturation	Annealing	Elongation	Repetition	Final extension	Yield	Control
NDM	94ᵒC for 30 sec	61.6ᵒC for 30 sec	72ᵒC for 1min	34 cycles	72ᵒC for 7 min	660bp	Dr. S.S (MDR-2014)
KPC	94ᵒC for 30 sec	50ᵒC for 30 sec	72ᵒC for 1min	34 cycles	72ᵒC for 7 min	863bp	ATCC-1705
OXA-1	94ᵒC for 30 sec	61.7ᵒC for 30 sec	72ᵒC for 1min	34 cycles	72ᵒC for 7 min	464bp	BSI-11904
OXA-48	94ᵒC for 30 sec	60ᵒC for 30 sec	72ᵒC for 1min	34 cycles	72ᵒC for 7 min	389bp	
mcr-1	95ᵒC for 30 sec	58ᵒC for 30 sec	68ᵒC for 1min	30 cycles	72ᵒC for 5 min	309bp	m-441[(Col-R 2019 2056(Col-R 2019)]
mcr-5	95ᵒC for 30 sec	50ᵒC for 30 sec	72ᵒC for 1min 35sec	30 cycles	72ᵒC for 5 min	1644bp	
